# Feasibility and safety report on robotic assistance for cervical pedicle screw fixation: a cadaveric study

**DOI:** 10.1038/s41598-024-60435-6

**Published:** 2024-05-13

**Authors:** Seungjun Ryu, Byeong-Jin Ha, Sunjin Yoon, Chang Kyu Lee, Dong Ah Shin, Keung-Nyun Kim, Seong Yi

**Affiliations:** 1 Department of Neurosurgery, Daejeon Eulji University Hospital, School of Medicine, Eulji University, Daejeon, South Korea; 2 Department of Neurosurgery, Hanyang University Guri Hospital, 153 Gyeongchun-ro, Guri, Gyeonggi-do, 11923 Republic of Korea; 3 Department of Neurosurgery, Spine and Spinal Cord Institute, Severance Hospital, Yonsei University College of Medicine, 50 Yonsei-ro, Seodaemun-gu, Seoul, 120-752 South Korea; 4 IBS Center for Cognition and Sociality, Expo-ro, Doryong-dong, Yuseong-gu, Daejeon, South Korea

**Keywords:** Biotechnology, Medical research, Engineering

## Abstract

This cadaveric study aimed to evaluate the safety and usability of a novel robotic system for posterior cervical pedicle screw fixation. Three human cadaveric specimens and C2-T3 were included. Freshly frozen human cadaver specimens were prepared and subjected to robot-assisted posterior cervical pedicle screw fixation using the robotic system. The accuracy of screw placement, breach rate, and critical structure violations were evaluated. The results were statistically compared with those of previous studies that used different robotic systems for cervical pedicle screw fixation. The robotic system demonstrated a high accuracy rate in screw placement. A significant number of screws were placed within predetermined safe zones. The total entry offset was 1.08 ± 0.83 mm, the target offset was 1.86 ± 0.50 mm, and the angle offset was 2.14 ± 0.77°. Accuracy rates comparable with those of previous studies using different robotic systems were achieved. The system was also feasible, allowing precise navigation and real-time feedback during the procedure. This cadaveric study validated the safety and usability of the novel robotic system for posterior cervical pedicle screw fixation. The system exhibited high precision in screw placement, and the results support the extension of the indications for robot-assisted pedicle screw fixation from the lumbar spine to the cervical spine.

## Introduction

The advent of robotic technology has revolutionized numerous fields, including neurosurgery, particularly spinal surgery^1–3^. The precision, accuracy, and consistency offered by robotic assistance have the potential to significantly improve surgical outcomes, particularly in complex procedures such as cervical pedicle screw fixation^4,5^. However, adopting this technology is contingent upon a rigorous evaluation of its feasibility and safety^4–6^.

Cervical pedicle screw fixation provides greater mechanical stability than lateral mass screw fixation^7^ but poses risks due to the challenging nature of the procedure and the unique, intricate anatomy of the cervical spine^4^. Accurate screw placement must be maximized to prevent neurovascular complications. In fact, a deviation of just a few millimeters can result in serious complications, such as nerve root, spinal cord, or vertebral artery injury^8,9^. Anatomical considerations for cervical pedicle screw placement include the mean distance between the pedicle and the inferior nerve root for all specimens, which ranges from 1.0 to 2.5 mm, and the distance between the medial pedicle and the dural sac, which ranges from 2.4 to 3.1 mm^10^.

Additionally, the presence of a high-riding vertebral artery at the C2 level, where the vertebral artery occupies the superior portion of the transverse foramen, can increase the risk of vertebral artery injury during pedicle screw placement if there is a misplacement of even a few millimeters^11^.

Robot-assisted surgical systems have emerged as a promising alternative to minimally invasive cervical pedicle screw fixation procedures guided by fluoroscopy, for which malposition rates of 6.7% to 29.1% have been reported^12–16^. These robotic systems provide an array of benefits, such as improved accuracy, reduced radiation exposure, and potentially superior clinical outcomes. However, they also present limitations like extended operative times and the necessity for intraoperative fluoroscopic confirmation and expensive O-arm preparations^4,5,17,18^.

While prior reports suggest that the accuracy of C-arm-based robot-guided surgery might be insufficient compared to that of O-arm-based systems, purchasing an O-arm is not economically feasible in many developing countries.

This study aimed to bridge the gap in the clinical field regarding the utility of the C-arm-based, economically efficient novel spine robotic system to expand indications from lumbar to cervical pedicle screw fixation by applying artificial intelligence for image data processing and registration based on the C-arm, which could potentially enhance accuracy.

With human trials yet to be authorized, we conducted cadaveric research to explore this hypothesis and compared our research data with previously published clinical data. This study provided valuable insights into the ongoing discourse on the role of robotic assistance in posterior cervical spinal surgery and its clinical validation.

## Materials and methods

### Specimen preparation

Three freshly frozen human cadaveric specimens from the C2-T3 region were used. All specimens underwent thorough visual inspection to ensure the absence of fractures, deformities, prior surgery, or severe spondylosis.

A CT scan was performed for all specimens (120 kV, 20 mA, 0.62 mm; GE Brightspeed, Boston, MA) to assess bone quality and obtain measurements for planning the ideal implant size and pedicle screw trajectory.

This study was approved by the Yonsei University Health System Ethics Committee IRB ('Single-institution, prospective researcher-led exploratory clinical study to evaluate clinical efficacy and safety of CUVIS-SPINE, navigation medical stereotaxic device' 1-2020-0025). The clear source of cadavers were adhered in the laboratory of the Korean Surgical Robot Education Center, through the Department of Anatomy at Yonsei University College of Medicine.

### Surgical instrumentation and technique

**Figure 1:**
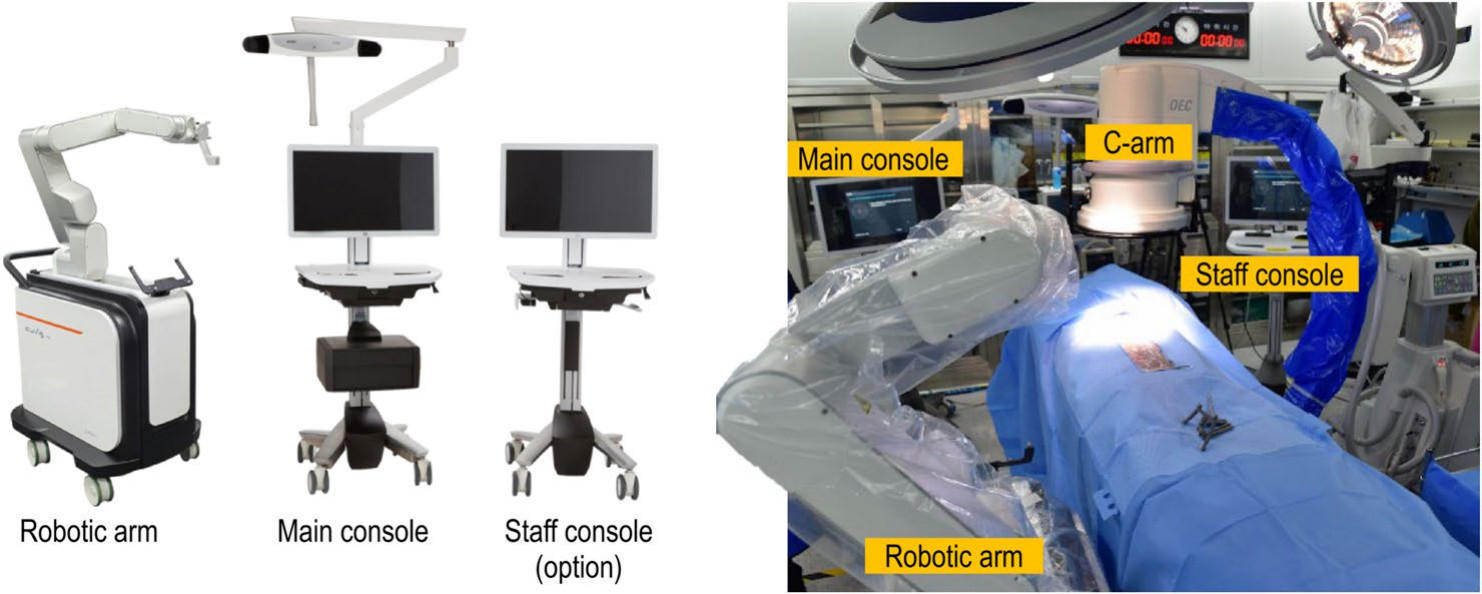
The CUVIS Spine surgical robot system is integrated with a surgical robotic arm, a main console with optical tracking devices, and a control and display workstation.

The cadaveric specimen was placed in the prone position on the surgical table, with the head stabilized using a Mayfield head clamp (MHC, manufactured by. Ohio Medical Instrument Co., Cincinnati, Ohio). The lower cervical spine was maintained in a neutral position. The surgical procedure involved using the CUVIS Spine robot developed by CUREXO and a long-arm screw manufactured by GS Medical. The necessary equipment for surgery included an intraoperative C-arm scan combined with CUVIS Spine navigation and robotic assistance provided by CUREXO (Seoul, Korea). The operating room settings are shown in Fig. 1.

The posterior neck area was prepared meticulously, including shaving, scrubbing, and draping, to ensure a sterile environment. The patient reference array was securely attached to a two-pin fixator, which was connected to a Mayfield holder and positioned adjacent to the surgical field. The CUVIS Spine Robotic System was positioned on the left side of the operating room table. Once the C-arm scan was completed in both the anteroposterior and lateral views with minimal motion artifacts, the captured images were automatically transferred to the CUVIS Spine navigation system for digital reconstruction and registration using preoperative spine CT scans.

Navigation tools were integrated into the system with types of arrays which attached at robot body and arm, and a navigated probe was used to determine the optimal entry point for the procedure. A median skin incision was made to expose the spinous process and laminae at the target level. The CUVIS Spine Robotic System was aligned and securely fastened along the desired trajectory for screw placement. Utilizing a 1.5-mm diameter navigated drill guided by image assistance, a pilot hole was created in the dorsal cortex of the left lateral mass through the cannula attached to the robotic arm. Tapping and insertion of the Long-arm Pedicle Screw System (GS Medical (23263 Madero Suite C Mission Viejo, California 92691, US)) were performed through the pedicle.

Screw diameter and length were determined perioperatively with the assistance of image guidance under the supervision of a senior surgeon. The same procedure was performed on the patient’s right side. A subsequent C-arm scan was performed intraoperatively to assess implant positioning. Upon confirmation of satisfactory implant positioning, the system was securely locked, and any attachments, such as the long-arm wing, were removed using a wing holder.

**Figure 2:**
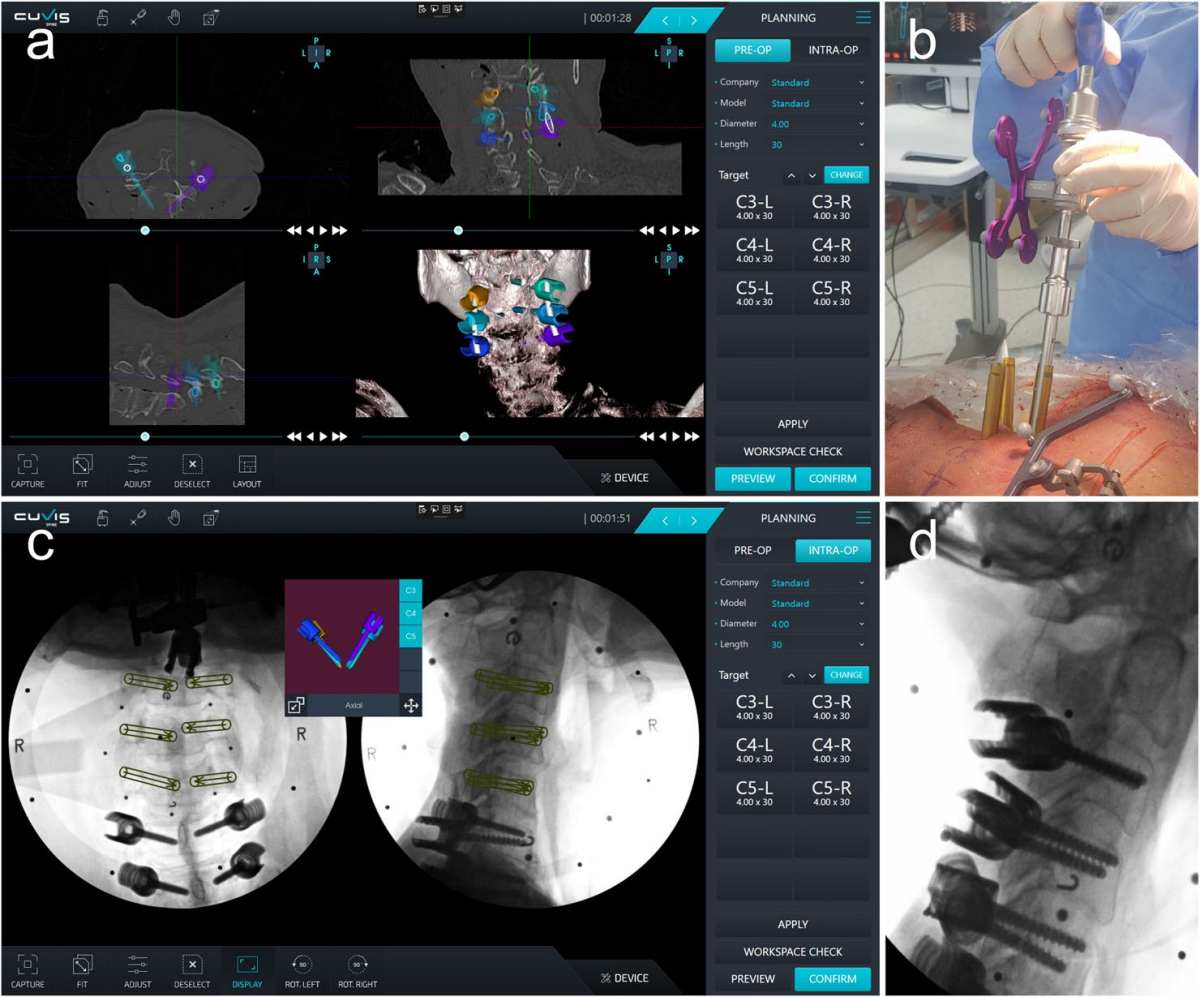
Procedures of robot-assisted posterior cervical pedicle screw insertion. (**a**) Preoperative CT-based planning of pedicle screw trajectory. (**b**) Robot-assisted pedicle screw placement. (**c**) Planned path overlaid on an intraoperative C-arm. (**d**) C-arm lateral image of cervical pedicle screw insertion.

These procedural steps, involving the use of the CUVIS Spine robot by CUREXO and the long-arm screw provided by GS Medical, in conjunction with advanced imaging and navigation technologies, enable precise and accurate fixation of posterior cervical pedicle screws. Intraoperative C-arm images were acquired using a C-arm scanner and were transferred to a workstation. Based on these images, the spine surgeon planned the entry point, angle, and depth of the pedicle screw and determined its characteristics (Fig. 2a). The planned path was overlaid on the intraoperative C-arm image, and the robotic arm was targeted to the planned path, allowing the surgeon to insert a screw (Fig. 2b–d). Integration of these technologies and techniques facilitates a minimally invasive approach that can improve patient outcomes and reduce surgical invasiveness.

### The specification of CUIVS-spine

CUVIS-spine consists of a robotic arm with a 5-degree-of-freedom manipulator, a main console including a 3D position measurement camera and a user monitor, and a staff console including an auxiliary monitor for the user. The manipulator consists of 2 to 5 axes, including 1 axis that performs linear motion in the forward and backward direction with 5 total degrees of freedom, and a rotation axis.

The Size and Weight were as follow. Robotic Arm : 602 × 648.5 × 1 072 (mm), 266 (kg), Main Console : 040–2340 × 650 × 728 (mm), 78 (kg), Staff Console : 1346 × 650 × 728 (mm), 32 (kg). Pose repeatability are position error ≤ 1.00 mm, orientation error ≤ 1.00°, and Pose accuracy are position error ≤ 1.00 mm, orientation error ≤ 1.00°. The Optical Tracking System (VEGA; NDI) and Target Tracking are operational.

### Accuracy evaluation

Postoperative CT images were used to assess the accuracy of pedicle screw placement in this cadaveric study involving 40 screws. In previous studies, the Gertzbein-Robbins classification system (GRS) was commonly employed to evaluate the accuracy of pedicle screw placement^12–15^. However, owing to the inherent differences in the spinal structure between the lumbar, cervical, and upper thoracic regions, applying the same criteria for accuracy evaluation in this study was challenging. Consequently, we adapted the Neo classification system for cervical pedicle screws to assess the accuracy of pedicle screw placement^16,19^. The evaluation involved categorizing the clinical grades into two groups: acceptable, defined as a grade of ≤ 1 for the Neo classification, and poor, indicated by a grade > 2 (Supplementary Fig.  S1).

**Figure 3:**
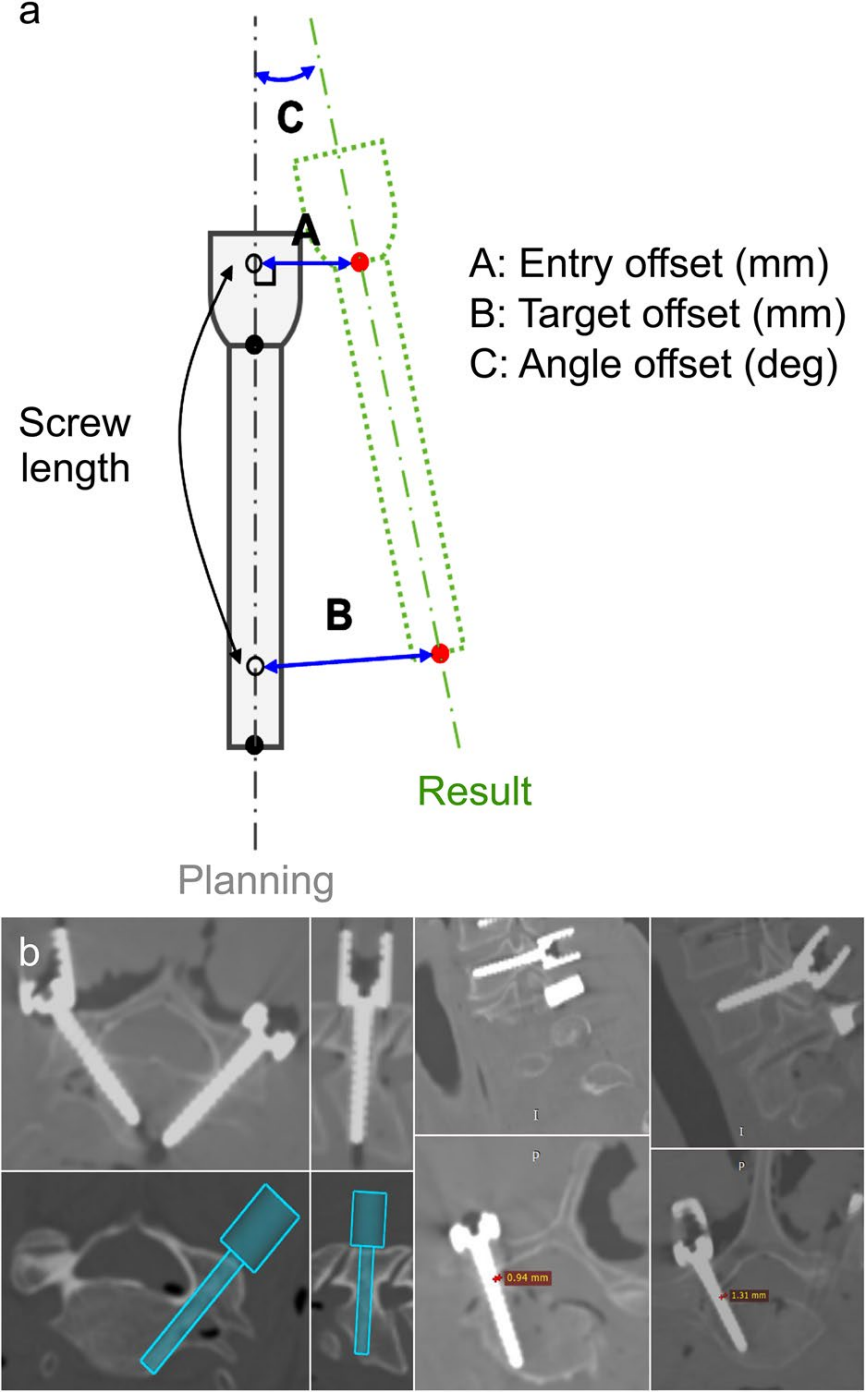
Measurement of the accuracy of robot-assisted posterior cervical pedicle screw insertion. (**a**) Definitions of three offsets, entry offset (mm), target offset (mm), and angle offset (deg.), between the planned and postoperative paths. (**b**) CT-based measurements of offset and cortical breach.

Although the GRS is useful, it lacks quantitative measures, and its limitations make comparing data across various experiments challenging. Consequently, there is a need for a quantitative measurement method that allows comparison with other datasets. To address this issue, the following evaluation criteria were utilized: entry offset, target offset, and angle offset, which were calculated based on postoperative CT images (Fig. 3).

Quantitative measurements were performed using CT scans for the 3D assessment of the accuracy of the screw tip, screw tail, and screw angulation. Image overlay analysis was performed to compare the planned trajectories prior to surgery with the actual post-surgery screw placement. The 3D accuracy assessment was documented for the anterior-most part of the screw (tip), posterior-most part of the screw (tail), and mean accuracy value. The angular offset was determined by calculating the difference in the angle between the vectors of the planned and placed screws^20^.

Additionally, the GRS, Neo, and Heary grades were used for postoperative accuracy analysis^19,21^. However, technical issues concerning file compatibility, trajectory file processing, and image analysis impede postoperative accuracy analyses in some instances. To ensure unbiased evaluations, all assessments were conducted independently by a blinded robotic engineer and neurosurgeon.

### Statistical analysis

Descriptive statistics for continuous variables are reported as mean ± standard deviation.

A comparative analysis was conducted juxtaposing the results of this study with those of previous studies that employed two distinct robotic systems for cervical and upper thoracic pedicle screw fixation. These studies functioned as comparative benchmarks for evaluating screw placement accuracy. The precision of screw placement was determined by calculating the percentage of screws positioned within predefined safe zones based on established guidelines and anatomical considerations. Identical criteria and parameters were applied to all three robotic systems to ensure consistency and comparability. To compare the accuracy grading between the domestically developed robotic system and the two previously studied systems, Fisher's exact test was conducted with the significance level set at p < 0.05. All statistical analyses were performed using SPSS version 26 (IBM Corp., Armonk, NY, USA).

### Ethics declarations

This study was approved by the Yonsei University Health System Ethics Committee IRB ('Single-institution, prospective researcher-led exploratory clinical study to evaluate clinical efficacy and safety of CUVIS-SPINE, navigation medical stereotaxic device' 1-2020-0025). The authors can confirm that all the methods employed in our research were carried out in full accordance with the appropriate guidelines and regulations in Scientific Reports.

### Approval for human cadaveric experiments

Approval for human cadaveric experiments was obtained through the Korea Surgical Robot Training Center and Yonsei University Health System Ethics Committee IRB.

### Consent to participate

The requirement for informed consent was waived by the Korea Surgical Robot Training Center and Yonsei University Health System Ethics Committee IRB since all participants in this study were cadavers.

## Results

In this study, three cadavers and 40 cervical pedicle screws ranging from C2 to T3 were analyzed. None of the cadavers had a history of preoperative spinal surgery or bony abnormalities. Intraoperative C-arm images were obtained, and if acquisition was hindered by individual anatomical variations, such as the jaw or shoulder, the corresponding level of the posterior cervical pedicle screw was excluded.

All procedures were performed using the CUVIS spine robot system, and postoperative CT scans were performed to analyze the screw path.

### Primary measures of robot-assisted pedicle screw insertion accuracy

Offset measurements were performed for 40 cervical pedicle screws inserted from C2 to T3 with the following distribution: C2 (2), C3 (2), C4 (4), C5 (6), C6 (6), C7 (6), T1 (6), T2 (4), and T3 (4). The entry offsets were as follows: 0.74 ± 0.83, 1.08 ± 0.83, 0.84 ± 0.45, 1.61 ± 0.79, 1.39 ± 1.05, 1.24 ± 0.77, 2.39 ± 0.40, 2.60 ± 0.60, and 0.74 ± 0.44 mm, with a total entry offset of 1.50 ± 0.94 mm. The target offsets were 0.51 ± 0.19, 0.30 ± 0.09, 0.92 ± 0.49, 2.45 ± 0.88, 1.86 ± 0.50, 1.74 ± 0.50, 1.78 ± 0.93, 1.41 ± 0.68, and 0.87 ± 0.36 mm, with a total target offset of 1.54 ± 0.87 mm. The angle offsets were 1.34 ± 0.25°, 1.64 ± 1.40°, 1.68 ± 0.93°, 2.75 ± 0.39°, 2.97 ± 0.85°, 1.71 ± 0.08°, 2.14 ± 0.77°, 2.11 ± 0.58°, and 1.62 ± 0.60°, with a total angle offset of 2.13 ± 0.88° (Table 1).

**Table 1:** Primary measures of robot-assisted pedicle screw insertion accuracy.

	Number of screws	Entry offset (mm)	Target offset (mm)	Angle offset (°)
C2	2	0.74 ± 0.83	0.51 ± 0.19	1.34 ± 0.25
C3	2	1.08 ± 0.83	0.30 ± 0.09	1.64 ± 1.40
C4	4	0.84 ± 0.45	0.92 ± 0.49	1.68 ± 0.93
C5	6	1.61 ± 0.79	2.45 ± 0.88	2.75 ± 0.39
C6	6	1.39 ± 1.05	1.86 ± 0.50	2.97 ± 0.85
C7	6	1.24 ± 0.77	1.74 ± 0.50	1.71 ± 0.08
T1	6	2.39 ± 0.40	1.78 ± 0.93	2.14 ± 0.77
T2	4	2.60 ± 0.60	1.41 ± 0.68	2.11 ± 0.58
T3	4	0.74 ± 0.44	0.87 ± 0.36	1.62 ± 0.60
Total	40	1.50 ± 0.94	1.54 ± 0.87	2.13 ± 0.88

### Gertzbein-Robbins Scale, Neo, and Heary classifications for cervical and thoracic pedicle screw placement

**Figure 4:**
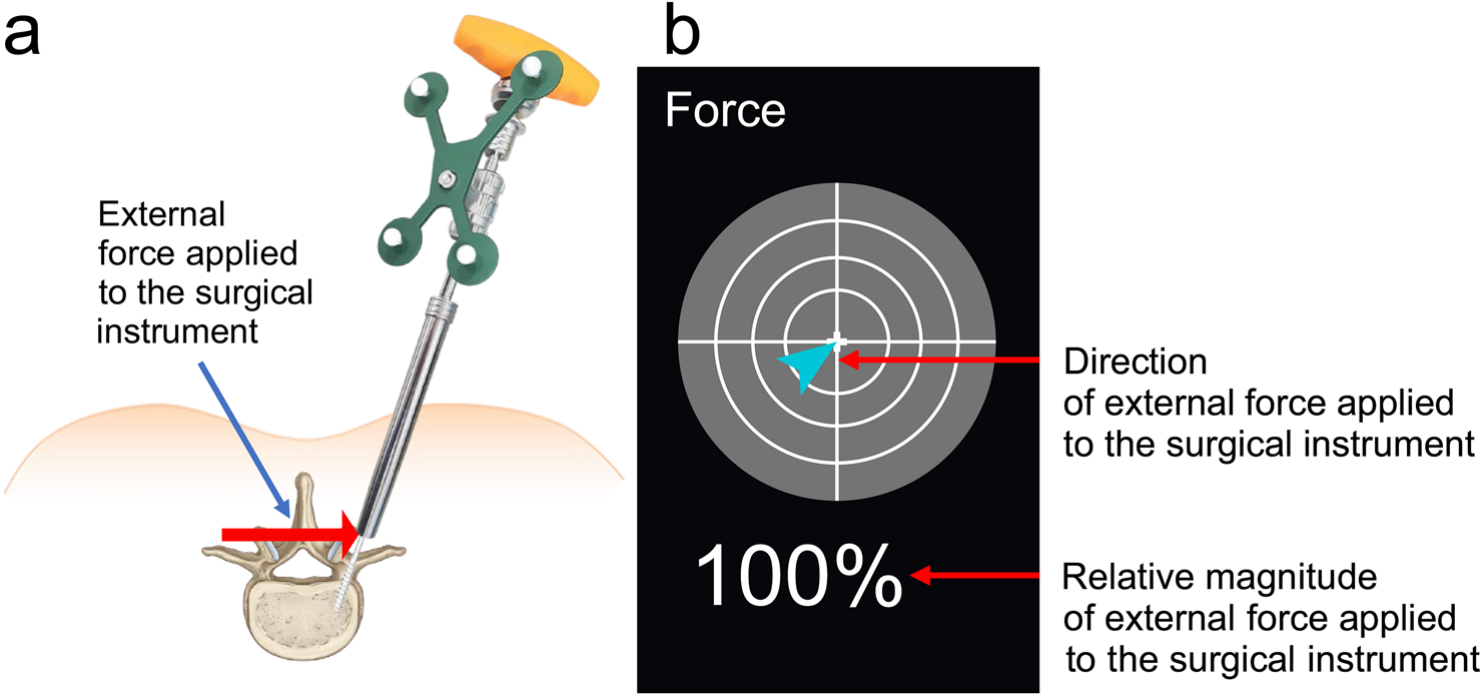
Graphical user interface (GUI) for direction and force during robot-assisted posterior cervical pedicle screw insertion. (**a**) Example of a situation where an external force is applied to the cervical pedicle screw instrument. (**b**) GUI portraying the direction of the external force applied to the instrument and the relative magnitude of force.

Of the 40 screws inserted from C2 to T3, 30 were classified as Grade A (75%), 9 as Grade B (22.5%), and 1 as Grade C (2.5%) according to the Gertzbein-Robbins Scale (GRS). In the Neo classification, 30 screws were Grade 0 (75%), nine were Grade 1 (22.5%), and one was Grade 2 (2.5%) (Supplementary Fig. S1). Additionally, according to the Heary classification, 30 screws were Grade I (75%), nine were Grade II (22.5%), and one was Grade IV (2.5%) (Table 2; Supplementary Table S1). The CUVIS Spine robot system proved to be feasible, providing interactive real-time feedback during the procedure through a graphical user interface that displayed the direction of the external force and its relative magnitude to the spine surgeons (Fig. 4).

**Table 2:** Gertzbein-Robbins Scale, Neo, and Heary classifications for cervical and thoracic pedicle screw placement.

Gertzbein-Robbins Scale						Neo classification					Heary classification				
	Number of screws	Grade A	Grade B	Grade C	Grade D		Grade 0	Grade 1	Grade 2	Grade 3		Grade 1	Grade 2	Grade 3	Grade 4
C2	2	2				C2	2				C2	2			
C3	2	2				C3	2				C3	2			
C4	4	4				C4	4				C4	4			
C5	6	4	1	1		C5	4	1	1		C5	4	1		1
C6	6	4	2			C6	4	2			C6	4	2		
C7	6	4	2			C7	4	2			C7	4	2		
T1	6	5	1			T1	5	1			T1	5	1		
T2	4	2	2			T2	2	2			T2	2	2		
T3	4	3	1			T3	3	1			T3	3	1		
Total	40	30	9	1	0	Total	30	9	1	0	Total	30	9		1

## Discussion

The implementation of pedicle-screw fixation for surgical interventions in the cervical region necessitates utmost precision due to potential complications^22,23^. This necessity has spurred extensive innovation and research, focused on enhancing the precision of insertion techniques using advanced modalities from O-arms to sophisticated robotic navigation systems^4,5,24^. Consequently, cervical pedicle screw insertion using robotics combined with a CT-based navigation system has been implemented in clinical trials and has received ministry approval in various countries, utilizing intraoperative 3D scan-based techniques^18^.

In light of these circumstances, we conducted a benchmark accuracy of cervical pedicle screw placement to compare the precision of our robotic system with others. Clinical grading of cervical and upper thoracic spine instrumentation using the CUVIS Spine robot system with intraoperative C-arm imaging and the digital reconstruction and registration method revealed that 39 (97.5%) were acceptable and one (2.5%) was poor. A comparative benchmark analysis demonstrated accuracy rates comparable to those of previous studies using different robotic systems, such as the TINAVI robot (TINAVI Medical Technologies, Beijing, China)^5^ and the Cirq robot (Brainlab AG, Munich, Germany)^4^ (Table 3). Fisher’s exact test showed a statistically significant difference among the three groups. Post-hoc analysis revealed that the CUVIS Spine robot system with intraoperative C-arm imaging was not statistically inferior to the TINAVI robot (TINAVI Medical Technologies, Beijing, China)^5^ or Cirq robot (Brainlab AG, Munich, Germany)^4^ with intraoperative 3D image-based systems in cervical pedicle screw fixation.All of the robotic systems in this study demonstrated a high accuracy rate in screw placement, with a substantial proportion of screws positioned within preestablished safe zones.

**Table 3:** Benchmark comparison of clinical grading-based acceptability rates with previous reports of.

	CUVIS spine	TINAVI robot^5^	Cirq robot^4^	*p*-value
Acceptable	39	175	24	0.015
Poor	1	5	4	
Total	40	180	28	

The general consensus is that the accuracy and safety of cervical pedicle screw placement using navigation systems with robotics are superior to those of conventional fluoroscopy-guided techniques^4,5,25,26^. Although the superiority of robotic systems using intraoperative O-arm-based or 3D scan-based images has been confirmed, the cost aspect must be considered because O-arms or 3D C-arms can be expensive. For widespread usage and cost-effectiveness, if a navigation system using a C-arm already owned by hospitals is available, more hospitals will be able to implement efficient and reliable surgical methods at an economic cost. Therefore, we developed a robot-assisted spine surgery system using a C-arm-based navigation system and expanded the indications from the thoracolumbar to the cervical region. This novel technology is continually improving with artificial intelligence algorithms and compiled data and may be particularly helpful in patients with challenging cervical anatomy.

Our study presents promising evidence of the feasibility and safety of robot-assisted posterior cervical pedicle screw fixation. The benchmark demonstrated that the high accuracy of cervical pedicle screw placement achieved with robotic assistance is consistent with previous studies^4,5^, highlighting the potential for the future development of this technology in comparison with cutting-edge robotic spine surgery techniques from other systems and countries. The CUVIS Spine robot system, which utilizes intraoperative C-arm imaging, is not inferior to the TINAVI robot (TINAVI Medical Technologies, Beijing, China)^5^ or Cirq robot (Brainlab AG, Munich, Germany)^4^, both of which employ intraoperative 3D image-based systems for cervical pedicle screw fixation. Using an algorithm that digitally reconstructs radiographs for application in 2D-3D image registration (Supplementary Fig. S2), the CUVIS spine robotic system achieves cervical pedicle screw accuracy similar to that of the TINAVI and Cirq robot systems.

Considering the unique anatomical features of the cervical spine, it is need to establish evaluation criteria for the measured data and define the novel acceptable ranges for deviations in distance and angle. However, the most of the previous studies demonstrated only GRS grade as the assessment of screw accuracy based on postoperative CT scans. Considering the findings reported by Godzik et al.^27^, which presented a mean 3D accuracy of 5.0 ± 2.4 mm, mean 2D accuracy of 2.6 ± 1.1 mm, and mean angular offset of 5.6° ± 4.3° across the total 70 screws of 17 patients and our accuracy results, we can parsimoniously suggest the acceptable ranges for these deviations have been defined based on the anatomical constraints and surgical objectives, an entry offset and target offset of less than 2 mm and an angle offset of less than 5° were considered with chance level. Further analysis of multi-center data for establish evaluation criteria for the measured data and define acceptable ranges for deviations in distance and angle or meta-analysis should be needed to make general consensus. The cervical region, unlike the lumbar, may necessitate heightened precision in screw placement to mitigate potential complications.

The results of our study indicate that the offset of robot-guided cervical pedicle screws is comparable to or even smaller than the offset observed in thoracolumbar clinical results^26–28^. This finding is particularly significant given the inherent anatomical and surgical differences between the cervical and thoracolumbar regions. The anatomical orientation of the entry point in the cervical spine is a key factor contributing to this outcome. Unlike the lumbar spine, where the entry point surface is typically slanted, the entry point of the cervical spine is anatomically perpendicular to the screw path. This perpendicular orientation reduces the likelihood of skiving, a common issue in the lumbar spine due to the lumbar facet-to-pedicle angle surface. Skiving or deviating the screw from the planned path can lead to inaccurate placement, potentially increasing the risk of complications.

Additionally, the entry point in the cervical spine is typically farther from structures such as the facet joints, which is a skiving risk factor for degenerative changes. Degenerative changes can lead to anatomical alterations that complicate pedicle screw placement. The relative distance of the entry point from these structures in the cervical spine compared with that in the lumbar spine may reduce the impact of degenerative changes on screw placement, further contributing to the smaller or comparable offset observed in our study.

However, this study has several limitations. Our study was constrained by its design, which utilized cadavers, a necessary step given the current regulatory environment in South Korea. Additionally, our study did not conduct a direct comparison with conventional fluoroscopy-guided techniques, such as those used in prospective controlled studies^25^, restricting the generalizability of our findings. Future studies should aim to validate our results in a clinical setting once robotic surgery for the cervical spine has been approved in South Korea.

Despite these challenges and limitations, the potential benefits of robot-assisted surgery in enhancing patient outcomes^29^ underscore the need for continued research and development in this field. Additional studies are required to confirm these results and investigate potential approaches for optimizing the accuracy and safety of robot-guided pedicle screw fixation from the thoracolumbar to the cervical spinal regions. Future advances should focus on resolving the existing limitations and improving the incorporation of robotic systems into surgical workflows.

## Conclusions

This study validated the safety and usability of a robotic system developed in Korea for posterior cervical pedicle screw fixation. The system exhibited high precision in screw placement, comparable to the results of previous studies. Although our research offers encouraging evidence supporting the feasibility and safety of robot-assisted cervical pedicle screw fixation, additional studies are necessary to corroborate these results and further investigate the potential advantages and drawbacks of robotic technology in cervical spinal surgery.

### Supplementary Information

Supplementary Information.

## Data Availability

The data supporting this study's findings are not openly available due to reasons of sensitivity and are available from the corresponding author upon reasonable request.
