# Supplementary material for: Feasibility and safety report on robotic assistance for cervical pedicle screw fixation: a cadaveric study
Source: Sci Rep. 2024 May 13;14:10881. doi: 10.1038/s41598-024-60435-6 (PMC11091198; doi:10.1038/s41598-024-60435-6)
Supplement: Supplementary file 1 — Supplementary Information. [file 41598_2024_60435_MOESM1_ESM.docx]

**Supplementary Material**


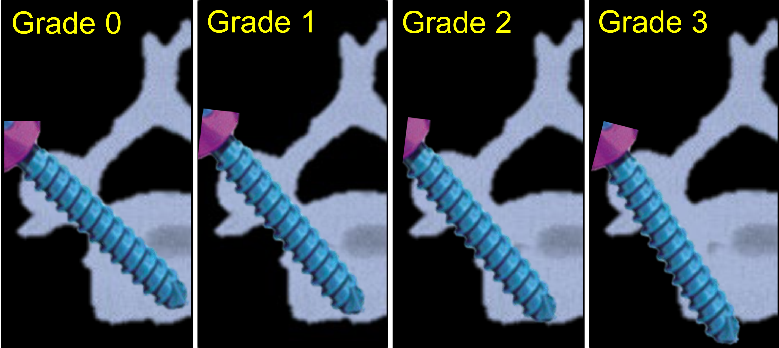


**Supplementary Figure S1.** Neo classification of pedicle perforation in the cervical spine

Grade 0: no perforation, Grade 1: perforation < 2 mm, Grade 2: perforation > 2 and < 4 mm, Grade 3: perforation > 4 mm

**Reference**

Yao, Xiang, and Shiqing Liu. "In vitro study of accuracy of subaxial cervical pedicle screw insertion using calipers based on the gravity line." Plos one 12.7 (2017): e0181324.

**
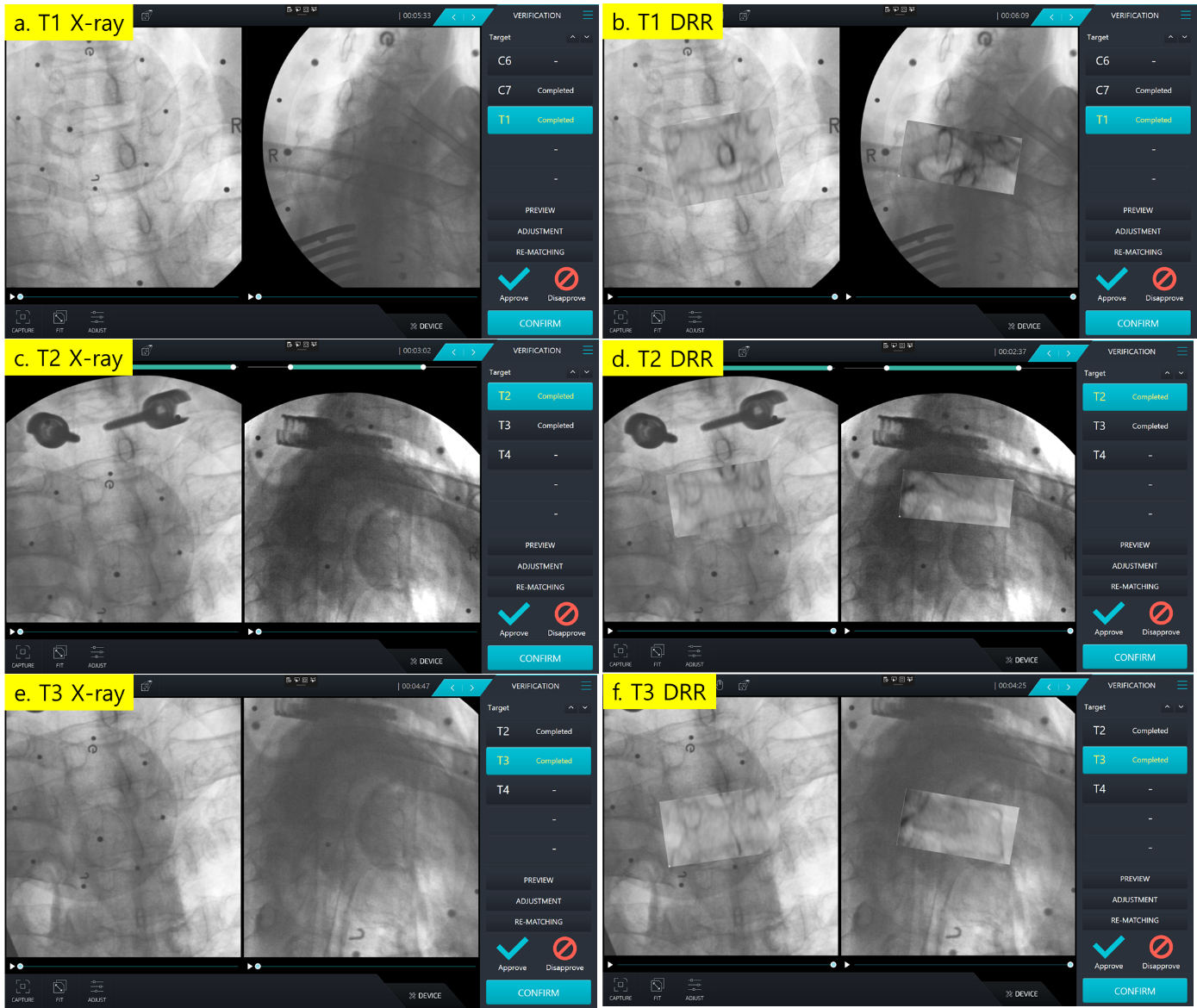
**

**Supplementary Figure S2**: Enhanced Visualization Process Through Overlay Technique

The overlay technique employed to enhance the visualization of anatomical structures (T1, T2, T3) from C-arm lateral radiographs (X-ray images : a, c, e) to digitally reconstructed radiograph (DRR: b, d, f) images.

**Supplementary Table S1.** Heary classification grade and breach explanations

| Grade | Breach |
| --- | --- |
| 1 | None |
| 2 | Lateral, but screw tip is within VB |
| 3 | Anterior or lateral breach of screw tip |
| 4 | Medial or inferior breach |
| 5 | Breach that required immediate revision (due to proximity to sensitive structures) |

**Reference**

Heary, R. F., Bono, C. M., & Black, M. Thoracic pedicle screws: postoperative computed tomography scanning assessment. J. Neurosurg. 100, 325-331 (2004).
